# The prevalence of low back pain in the emergency department: a descriptive study set in the Charles V. Keating Emergency and Trauma Centre, Halifax, Nova Scotia, Canada

**DOI:** 10.1186/s12891-018-2237-x

**Published:** 2018-08-23

**Authors:** Jordan Edwards, Jill Hayden, Mark Asbridge, Kirk Magee

**Affiliations:** 1 Department of Community Health & Epidemiology, Dalhousie University, Halifax, NS Canada; 2 Department of Emergency Medicine, Charles V. Keating Emergency & Trauma Centre, Halifax, NS Canada; 3 Department of Epidemiology & Biostatistics, Schulich School of Medicine & Dentistry, Western University, London, ON Canada

**Keywords:** Low back pain, Emergency setting, Prevalence estimate, Policy decision maker

## Abstract

**Background:**

While low back pain is a common presenting complaint in the emergency department, current estimates from Canada are limited. Furthermore, existing estimates do not clearly define low back pain. As such, our main objective was to estimate prevalence rates of low back pain in a large Nova Scotian emergency department using various definitions, and to describe characteristics of individuals included in these groups. An additional objective was to explore trends in low back pain prevalence in our emergency department over time.

**Methods:**

We conducted a cross sectional analysis using six years of administrative data from our local emergency setting. We first calculated the prevalence and patient characteristics for individuals presenting with any complaint of back pain, and for groups diagnosed with different types of low back pain. We explored prevalence over time by analyzing presentation trends by month, day of the week and hour of the day.

**Results:**

The prevalence of patients presenting to the emergency department with a complaint of back pain was 3.17%. Individuals diagnosed with non-specific/mechanical low back pain with no potential nerve root involvement made up 60.8% of all back pain presentations. Persons diagnosed with non-specific/mechanical low back pain with potential nerve root involvement made up 6.7% of presentation and the low back pain attributed to secondary factors accounted for 9.9% of back pain presentations. We found a linear increase in presentations for low back pain over the study period.

**Conclusion:**

This is the first multi-year analysis assessing the prevalence of low back pain in a Canadian emergency department. Back pain is a common presenting complaint in our local emergency department, with most of these persons receiving a diagnosis of non-specific/mechanical low back pain with no potential nerve root involvement. Future research should concentrate on understanding the management of low back pain in this setting, to ensure this is the proper setting to manage this common condition.

**Electronic supplementary material:**

The online version of this article (10.1186/s12891-018-2237-x) contains supplementary material, which is available to authorized users.

## Background

Low back pain is one of the most common forms of musculoskeletal pain, prompting individuals to seek medical care [1, 2]. In 2002, low back pain was the fifth most common reason for all office based physician visits in the US [3]. A systematic review conducted by Dagenais et al., 2008 analyzed the total costs of low back pain to society and estimated that in the US the total costs - direct (medical and nonmedical), indirect costs, and intangible costs of low back pain - are between 84.1 billion and 624.8 billion US dollars annually [4].

Most individuals will develop low back pain at some point in their life, as the lifetime prevalence is between 49 and 90% [5]. It is currently accepted that the management of low back pain should begin in the primary care setting [6], and over half of visits for low back pain are to primary care physicians [5]. Nevertheless, a recent systematic review on the prevalence of low back pain in emergency settings [7] suggests that low back pain is a common presenting complaint to this setting (pooled prevalence estimate 4.3%). Results from the same systematic review [7] indicated that there are a number of gaps in the literature, particularly a lack of clear and detailed definitions of low back pain. Additionally, the review identified a need for studies comparing prevalence results from multiple definitions of low back pain and research conducted in Canada [7].

In this study, we addressed these gaps in the literature by conducting a cross sectional analysis, involving secondary use of data from a large emergency department in Nova Scotia, Canada. Our objectives were to estimate the prevalence of low back pain among patients presenting to the emergency department, using different definitions of low back pain, and to describe the characteristics of patients diagnosed with these distinct definitions of low back pain. Our secondary objective was to assess trends in low back pain prevalence in this emergency department over time.

## Methods

### Design and data sources

We conducted a cross-sectional analysis of emergency department administrative data collected between the 15th of July 2009 and the 15th of July 2015. All patients presenting to the emergency department were captured in the database.

### Emergency department setting

This study was conducted at the Charles V. Keating Emergency and Trauma Centre (QEII emergency department) in Halifax, Nova Scotia, Canada. It is a tertiary care teaching hospital and the largest emergency department in Atlantic Canada with approximately 71,000 patient presentations each year [8].

### Data collection

We collected data from the administrative database EDIS (Emergency Department Information System), which is the central information database used in the QEII emergency department. The database contains over one million patient records and offers access to these records in real time. The database is constantly updated with information about patients as they progress through the emergency department. EDIS is currently endorsed by the Canadian Association of Emergency Physicians, L’Association des Médecins d’Urgence du Quebec, the National Emergency Nurses Affiliation, the Canadian Paediatric Society and the Society of Rural Physicians of Canada [9].

We collected data on individuals as they passed through the emergency department. We collected data on patients’ presenting complaint codes, presenting level of pain, Canadian Triage and Acuity Scale (CTAS) scores and individuals’ time of arrival. Presenting complaints were captured using the EDIS presenting complaint list. Description of the CTAS scores can be found online in Additional file 1.

We gathered data on patient characteristics age, sex and whether patients currently had a primary care provider. Information on primary care providers was captured as a check box when individuals present to the emergency department. We also captured patients emergency department diagnosis using both ICD-9 and ICD-10 codes, as the QEII emergency department switched from the use of ICD-9 codes to ICD-10 codes between July 2012 and Feb 2013.

We collected data on patients’ length of stay in the emergency department, whether patients were admitted to hospital following the visit and the details of the type of emergency department visit (e.g. referred to the emergency department or transferred from another health facility). We also captured whether patients had repeat visits to the emergency department, who was responsible for payment in the emergency department (e.g. department of health or workers’ compensation) and whether the patient received any imaging services (x-ray, CT, MRI). A list of the characteristics captured can be found in Additional file 2.

### Study population

We defined our eligible population as all adults presenting to the emergency department, excluding patients’ deceased on arrival. Adults were defined as individuals over the age of 16 (the minimum age of intake in our emergency setting). We included patients who arrived to the emergency department independently or by emergency health services (ambulance or helicopter). The eligible population made up the denominator in our prevalence estimate. This included the total number of emergency department visits [10, 11] and the total number of individual patients presenting to the emergency department [12] over the study period.

### Low back pain definitions

We first explored patient presentations and patient characteristics for individuals presenting with a triage complaint of “back pain” or “traumatic back/spine injury”. These codes were used to capture individuals potentially diagnosed with serious or non-serious low back pain. From this population, we defined three clinically relevant low back pain patient groups based on patient’s emergency department discharge diagnostic ICD codes: 1. low back pain with no potential nerve root involvement, 2. low back pain with potential nerve root involvement and 3. low back pain with attributed to trauma or other secondary factors (see Additional file 3, Fig. 1). ICD diagnoses included in each group was determined by consultation of previous studies [13, 14] and consensus with three independent researchers, which included an emergency physician and a back pain content expert. In the case of disagreement, discussion between the three reviewers was used to reach consensus.

1. *Non-specific/mechanical low back pain with no potential nerve root involvement* was defined as low back pain not attributed to an identifiable specific pathology [2]. Non-specific low back pain is described as pain, muscle tension, or stiffness localized below the lower edge of the chest and above the upper thigh [15]. For example, we included patients assigned ICD codes 724.5 “back pain” and 847.2 “low back strain” in this group (Additional file 3). A more specific definition of low back pain with no potential nerve root involvement, excluding ambiguous codes (e.g. 715.90 “osteoarthritis”), was used for sensitivity analysis (Table 1).
2. *Non-specific/mechanical low back pain with potential nerve root involvement* was defined as low back pain that included neurological signs and symptoms. This included patients with low back pain including irritation/compression of a lumbar nerve root). For example, we included patients assigned ICD codes 724.3 “sciatica” and 729.2 “radiculopathy” in this group (Additional file 3).
3. *Low back pain attributed to secondary factors* defined patients presenting with low back pain who are diagnosed with another etiology, for which low back pain may be a symptom, and often requiring different and sometimes urgent care. For example, we included patients assigned ICD codes of 441.9 “aortic aneurysm” and 577.0 “pancreatitis” in this group (Additional file 3).

**Fig. 1:**
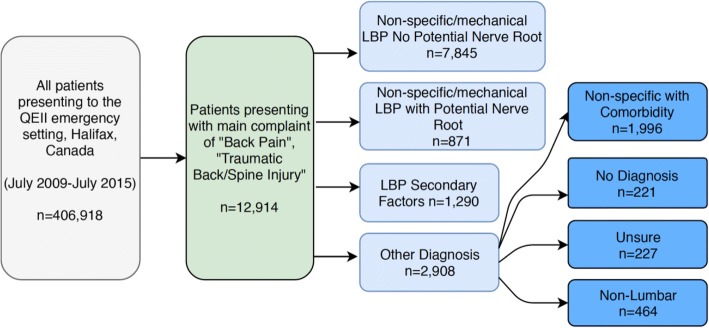
Flow diagram of the complete study population

**Table 1:** ICD-9/10 coding for a definition of low back pain that is representative of the literature

Description	ICD-9 Code
Myalgia	729.1
Muscle spasm	728.85
Mechanical Low Back Pain	724.2
Recurrent Low Back Pain	724.2
Back Pain	724.5
Chronic Back Pain	724.5
Pain-Back nyd	724.5
Muscle Spasm Back	724.8
Musculoskeletal Pain	729.1
Other msk	729.9
Chronic Pain (misc)	780.9
Pain nyd (Misc)	780.9
Lumbosacral Strain	846.0
Sprain Sacroiliac Int/Ligament	846.1
Low Back Strain	847.2
Other Sprain/ Strain Trunk	848.8
Description	ICD-10 Code
Myalgia	M79.1
Back Pain	M54.5
Muscle Strain	M62.6
Superficial inj Low Back / Pelvis uncomplicated	S30.80
Ow lower back / pelvis, uncomplicated	S31.0

Individuals presenting with a low back pain complaint, but not meeting the above definitions, were classified as ‘other’ and further classified for completeness based on independent researcher judgment. These groups were defined as *likely non-specific low back pain with comorbidity* (patients presenting with low back pain, but ultimately diagnosed with an etiology unlikely to have back pain as a symptom; consensus judgement that diagnosis was likely to be a co-morbid condition)*,* or *Non-lumbar back pain* (thoracic or cervical non-specific pain syndromes). Remaining patients with other diagnostic codes were classified as ‘unsure’.

### Analysis

We calculated the crude prevalence rates for all patients presenting with a complaint of low back pain, and for each of our defined low back pain groups. We performed a sensitivity analysis for the definition of non-specific/mechanical low back pain with no potential nerve root involvement by eliminating ambiguous ICD codes (see Table 1).

We described patient characteristics for each of our defined categories of low back pain. Frequencies and percentages were used to describe categorical variables. Continuous variables were described as means and standard deviations, or medians and inter-quartile ranges. Data was tested for normal distribution using the Shapiro-Wilk test. Means were used for variables with results that were normally distributed and medians were used for non-normally distributed data. Krustal-Wallis analysis of non-parametric data was used with a Bonferroni adjustment to test for significant differences between patient characteristics for separate definitions of low back pain. Significance was set at *p* = < 0.05.

Trends in low back pain prevalence over time were assessed using the available six-years of data grouped by month of presentation. The analysis of trend examines the low frequency variation in the data along with non-stationary changes in prevalence [16]. We fitted our data with a random walk model looking for seasonality by month. We used this model as we expect random presentations for back pain month to month [17]. The trend fitting our data was smoothed and tested for linearity using a linear regression. We performed these analyses for both prevalence estimates by month and presentations for low back pain per month. This allowed us to determine the trend in prevalence of low back pain with and without the influence of total presentations to the emergency setting. Due to partial data in the months of July 2009 and July 2015, we excluded these two months from the time series analysis.

We analyzed presentations by hour of the day and day of the week. We used density plots to explore presentations during separate hours of the day and days of the week and unpaired t-tests to test for significant differences between individuals presenting during work hours (Mon-Fri, 9 AM-5 PM) and non-work hours.

Significance was set at *p* = 0.05 level for all comparative analyses. Analyses were conducted using STATA IC 13.1.

## Results

There were a total of 406,918 presentations to the QEII emergency department during our six-year study period, of which 12,914 or 3.17% of individuals presented with a primary complaint of back pain, including “Back Pain” (12,706 presentations) and “Traumatic Back/Spine Injury” (208 presentations). The majority of patients (60.8%) presenting with back pain received a diagnostic code compatible with low back pain no potential nerve root involvement (overall prevalence of 1.93%). Individuals receiving a diagnostic code compatible with low back pain with potential nerve root involvement made up 6.7% of all back pain presentations (overall prevalence 0.22%); the low back pain attributed to secondary factors group accounted for 9.9% of all back pain presentations (overall prevalence 0.32%) (Fig. 1).

Characteristics of patients presenting to the emergency department with a complaint of back pain are described in Table 2. The median age of individuals was 45 (IQR: 30–60), and females made up 53.4% of the population. Patients spent a median length of 3.13 h (IQR: 1.93–5.1) in the emergency department and 34.7% of individuals presenting with back pain received x-rays.

**Table 2:** Patient characteristics of individuals presenting with a complaint of low back pain

Characteristic	Presenting complaint of LBP *n* = 12,914
Age, years (Median, IQR)	45 (30,60)
Female sex (#,%)	6897 (53.4)
CTAS (median, IQR))	4 (3–4)
Primary Care Provider (#,%)	12,211 (94.5)
Type of ED visit (#,%)	
Direct to Consult	310 (2.4)
Referral from GP	30 (0.2)
Return Visit	36 (0.3)
Missing	2247 (17.4)
Other (Emergency presentation)	10,291 (79.7)
X ray (#,%)	4478 (34.7)
CT (#,%)	968 (7.5)
MRI (#,%)	15 (0.12)
Hospital admission [#(%)]	878 (6.8)
Length of stay, hrs (Median, IQR)	3.13 (1.93–5.1)
Responsibility for payment (#,%)	
Department of Health, NS	10,680 (82.7)
Worker’s Compensation Board, NS	852 (6.6)
Other	1078 (8.3)
Missing	304 (2.4)

Note: *LBP* low back pain, *ED* Emergency Department, *HRS* hours, *CTAS* Canadian Triage and Acuity Scale, *IQR* Inter Quartile Range, *GP* General Practitioner, *NS* Nova Scotia

We compared patient characteristics between the three definitions of low back pain: low back pain no potential of nerve root involvement, low back pain with potential nerve root involvement and low back pain attributed to secondary factors (Table 3). We found that individuals with low back pain with no potential nerve root involvement had significantly higher CTAS scores (i.e. “less urgent”) than the other definitions of low back pain. Additionally, we found that low back pain with potential nerve root involvement had significantly higher CTAS scores compared to low back pain attributed to secondary factors. We also found that the low back pain with no potential nerve root involvement group had significantly lower age (median 43), compared to both the low back pain with potential nerve root irritation (median 46) and the low back pain attributed to secondary factors (median 58) groups. Furthermore, individuals with low back pain with no potential nerve root involvement were significantly less likely to be admitted to the hospital. Results of our Krustal-Wallis analysis are presented in Table 4.

**Table 3:** Patient characteristics of individuals presenting with a complaint of low back pain and diagnosed with various definitions of low back pain

Characteristic	Non-specific/mechanical LBP with No Potential Nerve Root Involvement *n* = 7845	Non-specific/mechanical LBP with Potential Nerve Root Involvement *n = 871*	LBP Attributed to Secondary Factors *n = 1290*
Age, years (Median, IQR)	43 (29,57)	46 (36,57)	58 (38,76)
Female sex (#,%)	4133 (52.7)	476 (54.6)	737 (57.1)
CTAS (median, IQR))	4 (3–4)	4 (3–4)	3 (3–3)
Primary Care Provider (#,%)	7411 (94.5)	825 (94.7)	1233 (95.6)
Type of ED visit (#,%)			
Direct to Consult	54 (0.7)	19 (2.2)	142 (11.0)
Referral from GP	12 (0.2)	2 (0.2)	5 (0.4)
Return Visit	19 (0.2)	6 (0.7)	5 (0.4)
Missing	1315 (16.8)	149 (17.1)	227 (17.6)
Other (Emergency presentation)	6445 (82.1)	695 (79.8)	911 (70.6)
Hospital admission [#(%)]	120 (1.5)	39 (4.5)	410 (31.9)
Length of stay, hrs (Median, IQR)	2.8 (1.8–4.4)	2.9 (1.7–4.9)	5.5 (3.5–9.2)
Responsibility for payment (#,%)			
Department of Health, NS	6364 (81.1)	751 (86.2)	1124 (87.1)
Worker’s Compensation Board, NS	31 (0.4)	47 (5.4)	28 (2.2)
Other	1292 (16.5)	55 (6.3)	95 (7.4)
Missing	158 (2.0)	18 (2.1)	43 (3.3)

Note: *LBP* low back pain, *ED* Emergency Department, *HRS* hours, *CTAS* Canadian Triage and Acuity Scale, *IQR* Inter Quartile Range, *GP* General Practitioner, *NS* Nova Scotia

**Table 4:** Results of Krustal-Wallis analysis used to test for significant differences between patient characteristics for separate definitions of low back pain (“non-specific/mechanical low back pain with no potential nerve root involvement”, “non-specific/mechanical low back pain with potential nerve root irritation” and “low back pain attributed to secondary factors”)

Characteristics	No Potential Nerve - Potential Nerve	No Potential Nerve - Secondary	Potential Nerve - Secondary
Age	<	<	<
	*p* < 0.001	*p* < 0.001	*p* < 0.001
Sex (More Females)	No difference	<	No difference
	*p* = 0.279	*p* < 0.001	*p* = 0.416
Length of stay	No difference	<	<
	*p* = 0.514	*p* < 0.001	*p* < 0.001
CTAS (Higher = less severe)	>	>	>
	*p* < 0.005	*p* < 0.001	*p* < 0.001
Hospital admissions	<	<	<
	*p* < 0.001	*p* < 0.001	*p* < 0.001

Our sensitivity analysis, which was used to test the robustness of our definition of low back pain with no potential nerve root involvement (eliminating ambiguous codes), resulted in an insignificant difference in prevalence (1.89%) compared to our non-specific low back pain estimate of (1.93%). Furthermore, we found no significant difference in age, sex or CTAS scores between both groups.

**Fig. 2:**
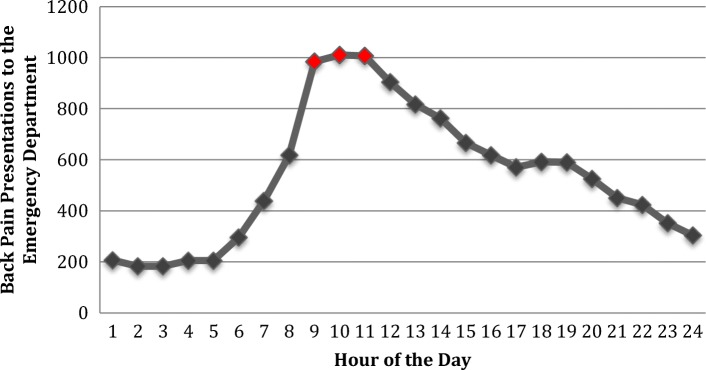
Patient presentations for back pain by the hour of the day. The analysis includes data from all days of the week. Peak hours of presentation were between 9 and 11 AM

**Fig. 3:**
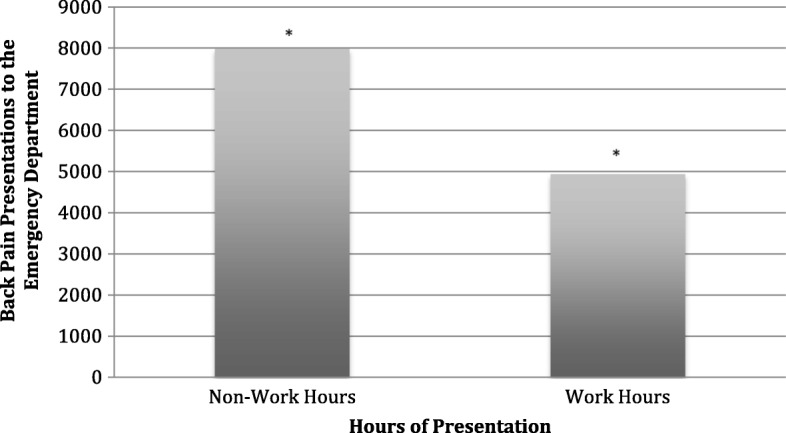
Patients presenting with low back pain during typical work hours, defined as 9 am to 5 pm Monday to Friday (38.2%) and non-work hours (61.8%) (*p* < 0.05)

**Fig. 4:**
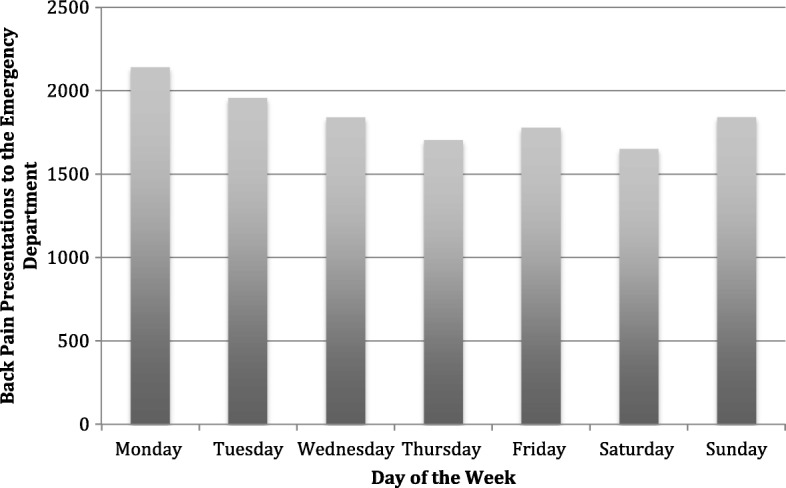
Presentations for back pain by day of the week

In our analysis of prevalence estimates over time, we found that peak hours for presentations for back pain were between 9 AM and 11 AM (Fig. 2). Our results indicate that significantly more individuals presented during non-work hours, 61.8%, compared to work hours (Fig. 3). Also, more persons presented on Mondays (16.6%) compared to all other days of the week (Fig. 4).

**Fig. 5:**
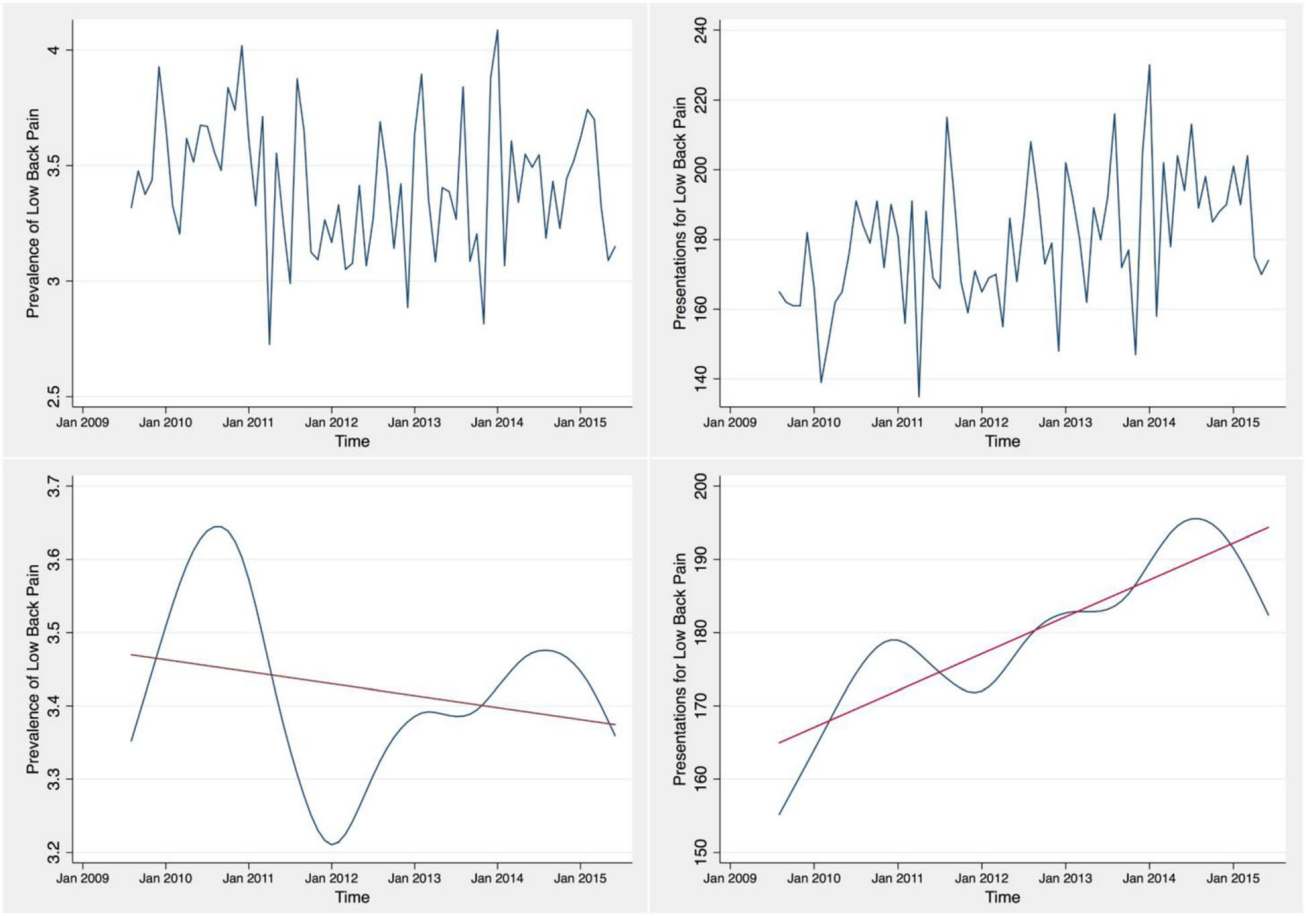
Prevalence and absolute number of presentations of persons with a complaint of “back pain” or “traumatic back/spine injury” between July 2009 and July 2015 grouped by month. The top panels display raw data and the bottom panels report the smoothed trend analysis with a linear regression. For our estimates of prevalence, the linear regression resulted in a slope of − 0.001 and an R^2^ value of 0.060. For our estimates of presentations, the linear regression resulted in a slope of 0.419 and an R^2^ value of 0.787

Our time series analysis showed that trends in the prevalence of low back pain in the emergency department remained stable over the six years of our study. The monthly prevalence of back pain ranged from 2.73 to 4.09%. There was no linear trend identified in the data; the linear regression resulted in a slope of − 0.001 and an R^2^ value of 0.06 (Fig. 5a).

Trend analysis for patient presentations for low back pain revealed a steady increase in patient presentations over the six years of data. The trend in presentations per month ranged from 135 to 230. The linear regression resulted in a slope of 0.42 with a R^2^ value of 0.78 (Fig. 5b).

## Discussion

Our multi-year study provides evidence that a substantial number of individuals, just over 3 %, present to the QEII emergency department with a complaint of low back pain. We found large variation in prevalence estimates for different definitions of low back pain. Most individuals presenting with back pain were diagnosed with low back pain with no potential nerve root involvement (overall prevalence 1.93%), while individuals with low back pain with potential nerve root involvement had an overall prevalence of 0.22% and individuals with low back pain attributed to secondary factors had an overall prevalence of 0.32%. These estimates are useful as they allow for comparison with other research in the field and they provide context for future prevalence estimates.

Our prevalence estimate for individuals presenting with back pain, 3.17%, is lower than what was observed in a meta-analysis of 16 prevalence studies of low back pain in the emergency department (4.3%) [7]. This difference may be due to the fact that the review included a broad spectrum of emergency settings, which may have different healthcare funding structures and access, and which may serve different patient populations.

Our results are comparable to other studies conducted in similar settings using similar back pain definitions of low back pain with no potential nerve root involvement and low back pain with potential nerve root involvement. For example, a study conducted in Canada [18], and one conducted in the US [13] reported prevalence estimates of 2.2%, and 2.3%, respectively, compared to our prevalence estimate of 2.15% (1.93% low back pain with no potential nerve root involvement and 0.32% low back pain with potential nerve root involvement).

To provide perspective, a study conducted in the US [19], which analyzed top presenting complaints, found that back pain (including neck pain), ranked as being the fifth most common presenting complaint in the emergency department [19]. Another recent analysis of Canadian emergency department visits, performed by the Canadian Institute of Health Information (CIHI), indicated that back pain is the sixth most common reason for an emergency department visit [20].

Studies using only ICD codes to quantify low back pain may be underrepresenting the burden of low back pain in emergency settings. Most studies in this field define prevalence for low back pain with and without a potential of nerve root involvement; however, other studies have not described prevalence of the low back pain attributed to secondary factors [7]. Including this group in prevalence estimates is important as it captures a clinically relevant population requiring serious intervention and significant resources. Future research should capture this population to increase the homogeneity of the literature and our understanding of the impact of the low back pain attributed to secondary factors group in various emergency settings.

This is one of the first studies to describe the prevalence and patient characteristics for groups of low back pain patients defined using discharge diagnostic codes. Results indicate that the severity of patients increases as our definitions progress from low back pain with no potential nerve root involvement to low back pain with potential nerve root involvement to low back pain attributed to secondary factors. This was reflected in our analysis of CTAS scores, which decreased with increasing severity of each definition of low back pain. This finding was both statistically and clinically significant. The findings strengthen our confidence and understanding of the severity of each of our definitions of low back pain, as they relate to the amount and urgency of care required for persons presenting with low back pain. We additionally found that for increasingly severe definitions of low back pain, length of stay increases, hospital admissions increase and so does median age of patients. We found that 27.4% of individuals diagnosed with low back pain with no potential nerve root involvement received x-rays. This result is similar to an analysis performed in the US [13], which found 30.5% of individuals received x-rays for back-related presentations to the emergency department. As we were not able to determine whether the x-rays were warranted, further analysis is required, and could be done by examining the prevalence of individuals presenting with a complaint of back pain along with red flag symptoms.

Our exploration of trends in low back pain presentations to the emergency department over time found that the prevalence of low back pain has remained relatively stable over the six years of the study period. However, there has been a steady increase in the number of presentations for low back pain over the past six years. This indicates that the emergency department has had a relative increase in total patient presentations, including back pain, over the past six years. The increase in emergency department and back pain patients may be due to changes in primary care availability, an increase in population or a decrease in population health. Further research is needed to understand this result, in addition to a broader exploration of the use of emergency settings to treat low back pain. A comparison between the treatment of low back pain in emergency settings and primary care settings would be useful to contextualize our findings, and provide insight into whether we should expect increases in presentations of low pain in emergency settings going forward.

### Strengths and limitations

A strength of this study was the use of a sensitivity analysis to explore the robustness of our definition of low back pain with no potential nerve root involvement. As we found insignificant differences between the two definitions (prevalence, patient characteristics), we can be confident in the robustness of our definition.

Our use of specific definitions of low back pain will benefit future research exploring the economic impact of back pain. As our separate definitions represent various levels of severity and intervention, they additionally represent different levels of economic impact. Our use of these definitions will provide a better picture of the economic burden of back pain in the emergency department.

We may be underestimating our prevalence estimate of low back pain, as we limited our study population to patients presenting with back pain. Because we used EDIS presenting complaint data to define our study population, our study does not include individuals who did not present with a complaint of back pain, however, left the emergency department with a diagnosis compatible with low back pain.

The accuracy of the presenting and diagnostic codes used in the emergency department administrative data (EDIS) is currently unknown. There may be differences between patient charts and what is recorded in the administrative dataset. The confidence in our results could be improved by performing a validity and reliability study on the EDIS database by comparing results from the database to patient charts [6].

Finally, the results of our study may not be generalizable to other parts of Canada, due to provincial differences in the population of patients seeking care for low back pain in the emergency department; for example socioeconomic status and the availability of emergency health services, as well as the structure of the health care system in Nova Scotia. We recommend that future research address this issue by analyzing prevalence in other emergency settings in Canada, including rural settings.

## Conclusions

Back pain is a common presenting complaint to emergency departments. Most individuals presenting with back pain are diagnosed with low back pain with no potential of nerve root involvement; however, we found that some individuals who present with back pain are discharged with other diagnoses. Moving forward, grouping patients using specific diagnostic codes would help us to better understand the prevalence of low back pain and its economic impact on the emergency department. Canadian research on the topic should include rural settings, where back pain is unexplored. In our local setting, future research should examine the increasing trend in presentations of low back pain and the impact of primary care service access on the prevalence of low back pain in the emergency department. We should also concentrate on understanding the management of low back pain in this setting, to ensure this is the proper setting and approach to manage this common condition.

## Additional files

Additional file 1: CTAS coding list. Describes how patients are classified based on the severity of their etiology upon arrival at our local ED. (DOCX 21 kb)

Additional file 2: Data dictionary. In our primary study, we collected the following information to describe the patient and health system characteristics from the EDIS database. (DOCX 18 kb)

Additional file 3: ICD-9/10 coding for definitions of low back pain: “Non-specific/mechanical low back pain with no potential nerve root involvement”, “Non-specific/mechanical low back pain with potential nerve root involvement” and “Low back pain attributed to secondary factors” based on results from the EDIS database. (DOCX 20 kb)

## Abbreviations

CIHI: Canadian Institute of Health Information
CTAS: Canadian Triage and Acuity Scale
EDIS: Emergency Department Information System
ICD: International Classification of Disease
US: United States
