# Supplementary material for: The prevalence of low back pain in the emergency department: a descriptive study set in the Charles V. Keating Emergency and Trauma Centre, Halifax, Nova Scotia, Canada
Source: BMC Musculoskelet Disord. 2018 Aug 23;19:306. doi: 10.1186/s12891-018-2237-x (PMC6106829; doi:10.1186/s12891-018-2237-x)
Supplement: Supplementary file 1 — CTAS coding list. Describes how patients are classified based on the severity of their etiology upon arrival at our local ED. (DOCX 21 kb) [file 12891_2018_2237_MOESM1_ESM.docx]

**Additional file 1:** CTAS coding list. Describes how patients are classified based on the severity of their etiology upon arrival at our local ED.

| **Triage**  **Level** | **Acuity**  **Level** | **Time to**  **Physician** | **Usual**  **Presentation** | **Sentinel Diagnoses** |
| --- | --- | --- | --- | --- |
| I | Resuscitation | Immediate | - Code arrest - Major shock - Shock states - Near-fatal asthma - Severe respiratory distress - Altered mental state (unconscious or delirious) | - Traumatic shock - Pneumothorax (traumatic or tension) - Facial burns with airway compromise Severe burns > 30% body surface area - Overdose with hypotension or unconsciousness AMI with complications (CHF or hypotension) - Status asthmaticus - Head injury (major or unconscious) Status epilepticus |
| II | Emergent | < 15 min | - Head injury (risk features with or without altered mental state) - Severe trauma - Altered mental state (lethargic, drowsy, agitated) - Signs of serious infection (purpuric rash, toxic) Allergic reaction (severe) - Chemical exposure (eyes) - Nontraumatic, visceral chest pain (with or without associated symptoms) - Vomiting or diarrhea, suspicion of dehydration - Overdose (but conscious) or drug withdrawal - Abdominal pain (age > 50 yr) with visceral symptoms - Sexual assault - GI bleeding with abnormal vital signs - CVA with major deficit - Severe asthma (peak expiratory flow rate <40%) Moderate or severe dyspnea - Acute vaginal bleeding (pain scale > 5 with or without abnormal vital signs) - Neonate (age <7 days) - Fever (age <3 mo), with rectal temp > 38.0°C - Acute psychotic episode or extreme agitation - Diabetic hypoglycemia or hyperglycemia - Headache, with pain scale 8–10/10 - Chemotherapy or immunocompromise - **Pain scale 8–10/10 (abdominal, costovertebral angle, back, eye)*** | - Head injury - Trauma involving multiple sites - Multiple rib fractures - Neck or spinal cord injury - Anaphylaxis - Alkaline or caustic ocular burns - AMI, unstable angina or CHF - Chest pain NOS - Gastroesophageal reflux - Unspecified drug or medicinal overdose - Abdominal aortic aneurysm Appendicitis, cholecystitis - GI bleeding with hypotension - CVA - Severe asthma or COPD - Croup - Spontaneous abortion - Ectopic pregnancy or rupture Epiglottitis, meningitis, sepsis - Acute psychotic episode, agitation or DTs - Diabetic ketoacidosis - Hypoglycemia, hyperglycemia or migraine Renal colic Keratitis |
| III | Urgent | < 30 min | - Head injury: alert with vomiting - Moderate trauma - Abuse, neglect or assault - Signs of infection - Mild or moderate asthma (peak expiratory flow rate >40%) - Mild or moderate dyspnea - Cheat pain with no visceral symptoms (sharp or MSK, no previous heart disease) - GI bleeding with normal vital signs - Acute vaginal bleeding with normal vital signs - Seizure (alert on arrival) - Acute psychosis with or without suicidal ideation - Pain scale 8–10/10 with minor injuries - **Pain scale 4–7/10 with headache, costovertebral angle or back pain*** - Vomiting and diarrhea (age < 2 yr) without dehydration - Dialysis problems | - Head injury - Anterior shoulder dislocation - Tibia or fibula fracture - Bimalleolar or trimalleolar ankle fracture - Pyelonephritis or sepsis - Asthma without status or COPD Bronchiolitis or croup - Pneumonia Unspecified chest pain NOS (MSK, GI, respiratory) - Uncomplicated GI bleeding - Spontaneous abortion - Seizure Acute psychosis with or without suicidal ideation Low back pain, strain (disk) Migraine |
| IV | Less Urgent | < 1 h | - Head injury: alert with no vomiting - Minor Trauma - Acute abdominal pain - Vomiting and diarrhea (age > 2yr) without   Dehydration   - Headache: not migraine, not sudden - Earache - Chest pain, minor trauma or MSK injury: no distress - Suicidal ideation or depression - Corneal foreign body - Minor allergic reaction - **Chronic back pain*** - URI symptoms - Pain scale 4-7/10 | -Head injury: alert with no vomiting  -Colles’ fracture  -Ankle sprain  -Appendicitis  -Cholecystitis  -URI  -Otitis media or otitis externa  -Chest pain NOS (MSK, GI, Respiratory)  -Gastroesophageal reflux  -Suicidal ideation or depression  -Urticaria  -Corneal foreign body  **-Low back pain or strain*** |
| V | Non-Urgent | < 2 h | - Minor trauma: not necessarily acute - Sore throat without respiratory symptoms - Diarrhea alone, without dehydration - Vomiting alone, with normal mental status and no dehydration - Menses - Minor symptoms - Chronic abdominal pain - Psychiatric complaints - Pain scale < 4/10 | - **Low back pain or strain*** - URI - Gastroenteritis - Vomiting - Disorders of menstruation - Dressing changes or cast changes - Constipation - Neurotic, personality and nonpsychotic mental disorders - superficial laceration(s) |
