# Supplementary material for: The prevalence of low back pain in the emergency department: a descriptive study set in the Charles V. Keating Emergency and Trauma Centre, Halifax, Nova Scotia, Canada
Source: BMC Musculoskelet Disord. 2018 Aug 23;19:306. doi: 10.1186/s12891-018-2237-x (PMC6106829; doi:10.1186/s12891-018-2237-x)
Supplement: Supplementary file 2 — Data dictionary. In our primary study, we collected the following information to describe the patient and health system characteristics from the EDIS database. (DOCX 18 kb) [file 12891_2018_2237_MOESM2_ESM.docx]

# **Additional file 2:** Data dictionary. In our primary study, we collected the following information to describe the patient and health system characteristics from the EDIS database.

| **Variable** | **Measurement** | **Source** |
| --- | --- | --- |
| Age | Measured in years. (Presented as mean age and age ranges (16-25, 26-35, 36-45, 46-55, 56-65, 66-75, 76+)). | EDIS |
| Sex | Measured as a nominal variable, where 0=female, 1=male. | EDIS |
| Responsibility for Payment | Measured as a categorical variable, where 1=OHIP, 2=WCB/WCB Hospital Staff, 3=Other Province, 4=Non-resident of Canada, 5=DVA/Federal Government/DIA/RCMP, 6=Uninsured Resident/Unknown | EDIS |
| Primary Care Provider | Present/Absent: Measured as a dichotomous variable, where 0=Does NOT have a Primary Care Provider, 1=Does have a Primary Care Provider | EDIS |
| Referral Source | Yes/No: Measured as a dichotomous variable, where 0=NOT Referred to ED by Physician or Health Professional, 1=Referred to ED by Physician or Health Professional. | EDIS |
| Method of Arrival (MOA) | Measured as a nominal variable, where 0= Independently, 1= Ambulance, 2= Helicopter. | EDIS |
| Time of presentation | Date, hour and minute of a patient’s arrival. We will analyze as a nominal variable in two ways. The first 0= not presenting during work hours and 1= presenting during work hours (8 AM to 5PM). The second 0= presenting on a weekend and 1= presenting on a weekday. | EDIS |
| Chief Complaint / Reason for visit | EDIS presenting complaint list (# 551 Back Pain, #552 Traumatic Back/ Spine Injury). | EDIS |
| CTAS Score | Measured on a CTAS 5-point scale of severity: Resuscitation, Emergent, Urgent, Less Urgent, Non-Urgent. | EDIS |
| Type of ED Visit | Measured as a categorical variable, where 1=Emergency Presentation, 2=Direct to Consult, 3=Return Visit-PLANNED, 4=Return Visit-UNPLANNED, 5=811 Referral, 6=Trauma Team, 7=Referral from GP/Clinic | EDIS |
| Presenting Level of Pain (Pain Scale) | Measured as a categorical variable, where 0=No Pain, 1=Mild Pain, 2=Moderate Pain, 3=Severe Pain.  To describe the pain intensity of patients presenting to the ED with LBP. Consistent with the NRS-11 scale (Numeric Rating Scale), where 0=No pain, 1-3=Mild Pain, 4-6=Moderate Pain, and 7-10=Severe Pain | EDIS |
| ED Diagnosis (Main Problem) | ICD-9/ 10 Codes | EDIS |
| Leave against medical advice (AMA) | Measured as a nominal variable, where 0= Patients left without being seen by a physician, 1= Patients were seen by a physician. | EDIS |
| Departure Time (Disposition Time) | Hour and minute of a patient’s disposition time. | EDIS |
| Admission | Measured as a nominal variable, where 0= Patient is sent home from the ED and 1= Patient is admitted into the hospital. | EDIS |
| Length of Stay (LOS) | Measured in (Hours / Minutes) from triage coding to diagnosis coding. | EDIS |
