# Supplementary material for: The prevalence of low back pain in the emergency department: a descriptive study set in the Charles V. Keating Emergency and Trauma Centre, Halifax, Nova Scotia, Canada
Source: BMC Musculoskelet Disord. 2018 Aug 23;19:306. doi: 10.1186/s12891-018-2237-x (PMC6106829; doi:10.1186/s12891-018-2237-x)
Supplement: Supplementary file 3 — ICD-9/10 coding for definitions of low back pain: “Non-specific/mechanical low back pain with no potential nerve root involvement”, “Non-specific/mechanical low back pain with potential nerve root involvement” and “Low back pain attributed to secondary factors” based on results from the EDIS database. (DOCX 20 kb) [file 12891_2018_2237_MOESM3_ESM.docx]

**Additional file 3:** ICD-9/10 coding for definitions of low back pain: “Non-specific/mechanical low back pain with no potential nerve root involvement”, “Non-specific/mechanical low back pain with potential nerve root involvement” and “Low back pain attributed to secondary factors” based on results from the EDIS database.

| **Non-specific/mechanical low back pain (no nerve root involvement)** | **Non-specific/mechanical low back pain with potential nerve root involvement** | **Low back pain attributed to secondary factors** | |
| --- | --- | --- | --- |
| ICD 9/10; Diagnosis | ICD 9/10; Diagnosis | ICD 9/10; Diagnosis | ICD 9/10; Diagnosis |
| 715.90 osteoarthritis  719.45 pain - hip nyd  719.49 polyarthralgia  720.2 sacroiliitiss  721.3 spondylosis lumbar spine  721.3 sacroiliac arthritis  721.90 arthritis back  721.90 osteoarthritis back  724.2 mechanical low back pain  724.2 recurrent low back pain  724.5 pain - back nyd  724.5 back pain  724.5 chronic back pain  724.6 pain buttock  724.6 pain sacrum  724.79 pain coccyx  724.8 muscle spasm back  724.8 facet joint syndrome  728.85 muscle spasm  729.1 musculoskeletal pain  729.1 fibromyalgia  729.1 myalgia  729.1 myofascial syndrome  729.9 other msk  780.9 chronic pain (misc)  843.8 strain gluteal muscle  843.9 sprain hip  844.8 strain hamstring  846.0 lumbosacral strain  846.1 sprain sacroiliac jnt/ligament  847.2 low back strain  848.8 other sprain/strain trunk  998.1 bruising (po)  M13.9 arthritis, unspecified  M25.5 joint pain  M54.5 back pain  M62.6 muscle strain  M79.1 myalgia  M81.9 osteoporosis  S30.80 superficial inj low back / pelvis  V71.8 normal exam  Z71.9 counselling / medical advice | 722.10 herniated lumbar disc  722.2 herniated disc (neuro))  722.6 degenerative disc disease  724.3 sciatica  728.9 weakness leg  729.2 neuralgia  729.2 radiculopathy  729.2 radiculopathy leg  782.0 paresthesia, nyd  M48.0 spinal stenosis  R20.8 paresthesias - numbness | 041.9 other bacterial  052.9 chickenpox  053.9 herpes zoster  053.9 shingles  153.9 colon ca  183.0 ovary ca  199.1 metastatic cancer  199.1 all other ca's  203.0 multiple myeloma  208.0 acute leukemia  300.81 somatoform disorder  324.1 abscess, spinal  336.9 cord compression (neuro)  336.9 cord compression  344.60 cauda equina syndrome  410.70 nstemi  411.1 angina unstable  411.1 acute coronary syndrome  413.9 angina-stable  415.1 pulmonary embolus  423.9 pericarditis  441.0 dissection aorta  441.9 aortic aneurysm  482.9 bacterial pneumonia  483 atypical pneumonia  485 bronchopneumonia  511.89 hemopneumothorax  535.00 acute gastritis  540.1 diverticular abscess  540.9 acute appendicitis  541 possible appendicitis  555.9 crohn's disease  558.9 gastroenteritis  560.1 ileus  560.9 small bowel obstruction  560.9 large bowel obstruction  562.10 diverticulosis  562.11 diverticulitis  564.0 constipation  566 perirectal abscess  567.9 peritonitis  569.49 other anorectal  569.9 other gi condition  574.20 biliary colic  574.20 cholelithiasis  575.0 acute cholecystitis  576.1 ascending cholangitis  577.0 pancreatitis  577.8 gallstone pancreatitis  584.9 renal failure acute  590.8 pyelonephritis  591 hydronephrosis  592.1 ureteral calculus  593.9 mass kidney  595.9 cystitis  599.0 urinary tract infection  599.33 urosepsis  599.7 hematuria  601.9 prostatitis  604.90 orchitis  805.2 compression fracture thoracic spine | 614.9 pelvic inflammatory disease  620.2 ovarian cyst  625.3 dysmenorrhea  625.8 pelvic pain nyd  626.2 menorrhagia  629.9 other pelvic organ problem  632 missed abortion  644.10 labour  682.2 abscess back  682.2 abscess perineum  682.5abscess buttock  685.0pilonidal abscess  686.9 other cellulitis/abscess  720.0 ankylosing spondylitis  726.5 bursitis hip  728.88 rhabdomyolysis  730.20 osteomyelitis  788.0 renal colic  805.2 fracture thoracic spine  805.4 compression fracture lumbar sp  805.4 fracture lumbar spine  805.6 fracture coccyx  805.6 fracture sacrum  807.00 fracture rib  807.00 fracture ribs  808.0 fracture acetabulum  808.2 fracture pubic rami  808.43 multiple pelvic fractures  809.0 other fracture spine/trunk  820.8 fracture hip  827.0 other fracture pelvis/leg  861.21 contusion lung  876.0 stab wound back/laceration back  969.9 o.d. drugs of abuse - other  A09.9 gastroenteritis  B00.9 herpes  C90.0 multiple myeloma  G06.1 intraspinal abscess and granuloma  I20.0 unstable angina  I21.9 acute myocardial infarction  I26.9 pulmonary embolism  I71.9 aortic aneurysm  K56.6 sbo/lbo bowel obstruction  K62.9 anal and rectal disorder  K63.9 intestinal disease, other  K80.8 biliary colic / cholelithiasis  K85.9 pancreatitis, acute  L05.0 pilonidal cyst with abscess  M45 ankylosing spondylitis  M46.4 discitis  N10 pyelonephritis  N17.9 acute renal failure  N23 renal colic  N39.0 urinary tract infection  N41.0 prostatitis, acute  S22.90 fx thoracic vertebra, closed  S27.30 contusion lung, no ow  S31.0 ow lower back / pelvis, uncomplicated  S32.0 fx lumbar vert, closed  S32.20 fx coccyx, closed  S33.1 Dislocated lumbar vertebra |
